# Comprehensive Analysis of Cutting-Force Components in Milling Using RQA: Effect of Edge Geometry and Process Parameters

**DOI:** 10.3390/ma18163768

**Published:** 2025-08-11

**Authors:** Marcin Płodzień, Łukasz Żyłka, Michał Wydra, Rafał Rusinek

**Affiliations:** 1 Department of Manufacturing Techniques and Automation, The Faculty of Mechanical Engineering and Aeronautics, Rzeszow University of Technology, W. Pola Str. 2, 35-959 Rzeszow, Poland; plodzien@prz.edu.pl; 2 Department of Applied Informatics, The Faculty of Mathematics and Information Technology, Lublin University of Technology, Nadbystrzycka 38, 20-618 Lublin, Poland; m.wydra@pollub.pl; 3 Department of Applied Mechanics, Mechanical Engineering Faculty, Lublin University of Technology, Nadbystrzycka 36, 20-618 Lublin, Poland; r.rusinek@pollub.pl

**Keywords:** milling, serrated edge, cutting force, recurrence analysis, RQA

## Abstract

This study investigates the influence of cutting edge geometry (continuous, serrated, and wavy) and selected machining parameters (cutting speed *v_c_*, feed per tooth *f_z_*, and radial infeed *a_e_*) on cutting-force components and dynamic behavior during the milling of an AlZn5.5MgCu aluminum alloy. The analysis was based on box plots and Recurrence Quantification Analysis (RQA) applied to the cutting-force signal. The results demonstrated that serrated and wavy-edge tools generated significantly lower values of the normal force component F_fN_—up to −57% on average—compared to the continuous-edge tool, particularly at lower *f_z_* and *v_c_*, indicating enhanced process dynamics. At higher *a_e_* values, however, these tools induced increased signal variability—up to 300% greater—suggesting potential resonance excitation. RQA indicators, such as DET, Lmax, and LAM, revealed a strong dependence of system dynamics on tool edge geometry. Linear Discriminant Analysis (LDA) confirmed that RQA measures effectively distinguish between cutting-edge types. The study concludes that tooldge geometry substantially affects milling process stability and can be purposefully selected to optimize performance under varying machining conditions.

## 1. Introduction

Milling is one of the most commonly used material removal processes in the manufacturing of machine components. It is widely employed for the production of various mechanical parts across multiple industrial sectors, including tooling, energy, automotive, and aerospace industries. The continuous drive to reduce production times while simultaneously improving the dimensional accuracy of machined components fosters ongoing development in both cutting tools and milling strategies. This is particularly important in complex milling operations, such as high-performance machining of closed and constrained profiles, such as for example, pockets. In such cases, the large volumes of material removed can result in the formation of sizable and heavy chips, the evacuation of which from the machining zone becomes increasingly difficult [1].

**Figure 1:**
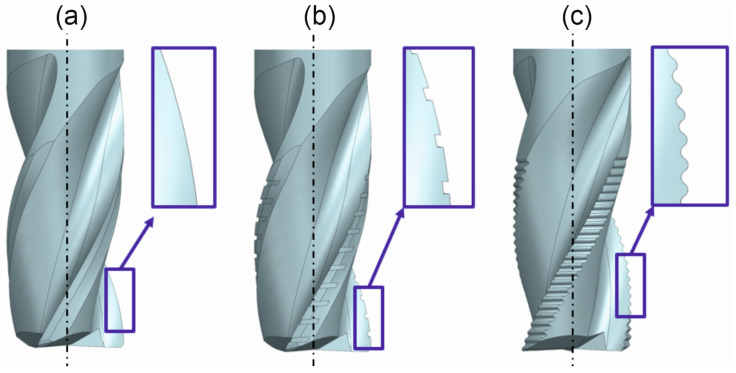
Type of cutting-edge profiles: (**a**) continuous, (**b**) serrated, (**c**) wavy.

One of the key approaches to optimizing the geometry of milling tools used in high-performance machining involves the appropriate design of the cutting-edge profile. Two primary alternatives are commonly employed in place of a continuous cutting edge: a wavy or an serrated edge (Figure 1). These geometries effectively divide the cutting edge into smaller segments, resulting in an increased number of discrete cutting tips. Consequently, the chip is segmented into smaller fragments, which significantly improves chip evacuation from the cutting zone, thereby enhancing process safety [2]. However, segmenting the cutting edge leads to an increase in the number of individual cutting interactions along the tool’s edge. This, in turn, results in a higher frequency of tool–workpiece contacts and has a considerable impact on the dynamics of the cutting process. Specifically, the excitation frequency increases and approaches the natural frequencies of the system components involved in the milling process. For this reason, the dynamic behavior of milling with serrated cutting edges remains a subject of ongoing research and analysis.

An essential aspect of milling cutters is the design of the cutting-edge geometry. Numerous studies have been conducted to investigate the influence of serrated and wavy cutting edges on the milling process. Koca and Budak optimized the geometry of serrated tools to reduce energy consumption and enhance cutting stability [3]. Their results demonstrated that an appropriately designed geometry can significantly increase the allowable depth of cut by as much as 60%. Hosseini et al. developed a CAD/CAM-based model for simulating milling processes using serrated tools [4,5]. They analyzed force distribution and chip thickness based on the geometrical parameters of the tool. Similarly, Grabowski et al. proposed a mathematical model for determining cutting forces and stability limits in milling with complex tool geometries, including serrated cutters [6]. Guo et al. also focused on modeling cutting forces and developed a force model for tools with wavy cutting edges [7]. The model incorporated the actual trajectory of the cutting edges in relation to the wave profile and analyzed the temporal variation in the cutting forces. It was demonstrated that both the wavelength and amplitude significantly influence the peak cutting-force values. Merdol, in his work, developed a comprehensive geometric, dynamic, and force model for tools with wavy edges [8]. His model considered the effects of chatter, torque, cutting forces, and power consumption. Sultan and Okafor investigated the influence of geometric parameters of wavy-edged tools on cutting forces during the milling of Inconel 718 under MQL (Minimum Quantity Lubrication) conditions [9]. The results showed that the highest forces occurred in the feed direction and that their magnitude increased with greater wave amplitude, but decreased with an increasing wave length and helix angle. Kountanya and Guo also developed a geometric and force model for serrated milling cutters, which was validated experimentally, confirming the model’s predictive capability [10].

The dynamics and stability of the milling process using serrated cutters have been the subject of extensive research efforts [11]. Machining dynamics play a critical role in determining the condition of the cutting tool, the machine tool structure, and the surface quality of the machined workpiece. In extreme cases, instability of the cutting process may occur, leading to chatter and degraded performance. Tehranizadeh et al. studied the effect of wavy profile shape (sinusoidal, trapezoidal, and circular) on cutting forces and process stability [12,13]. A dynamic milling model and semi-discretization method were used to analyze stability. The results indicated that proper selection of the wave shape can reduce cutting forces by approximately 30% and significantly increase the allowable depth of cut. Dombovari et al. explored the dynamic behavior and mechanics of milling with wavy-edged cutters [14]. Their study showed that such tools require lower driving torque and enable milling at greater depths of cut compared to standard cutters. Experimental results confirmed that wavy-edged cutters significantly expand the range of stable cutting parameters, thereby enabling more efficient machining. Similar conclusions were obtained by Burek et al., who investigated the influence of cutting-edge geometry on the cutting force in the High Performance Cutting (HPC) of an AlZn5.5MgCu aluminum alloy [15]. The results showed that serrated and wavy tools generated approximately 25% lower cutting forces compared to tools with continuous edges. Also Bari et al. proved that in aluminum machining, milling cutters with helical–circular (skewed–circular) profiles produced lower cutting forces than standard tools. However, both tool types exhibited similar stability characteristics in the presence of self-excited vibrations (chatter) [16]. The conducted research proves that the use of tools with serrated or wavy cutting edges has a significant impact on the milling process, particularly on the cutting force, and thus on the overall process dynamics.

One of the tools commonly used to analyze the dynamics and stability of machining processes is recurrence analysis [17,18]. This method typically involves the evaluation of Recurrence Quantification Analysis (RQA) measures based on signals acquired during machining, such as cutting-force components, acoustic emission, vibrations, and cutting power [19,20,21,22,23,24]. Numerous studies have addressed the issue of milling process stability and dynamics using recurrence-based methods. Litak et al. examined the relationship between Recurrence Quantification Analysis (RQA) measures and the dynamics of milling composite materials [22]. In their study, spindle speed—representing the system’s excitation frequency—was treated as a variable. The results indicated that only the DET measure is suitable for assessing the dynamics of the milling process. In a related study, Elias employed Cross Recurrence Quantification Analysis (CRQA) to evaluate the stability of a turning process based on spindle power signals [25,26]. He tested six CRQA metrics and demonstrated that changes in their values correlated with the onset of vibrations. Lajmert et al. investigated the use of cutting-force components for detecting the occurrence of chatter in the milling of Inconel alloys, employing recurrence-based analysis [27]. Rusinek [18] studied the applicability of RQA indicators for assessing milling stability in aluminum alloys. He demonstrated that five recurrence measures—DET/RR, LAM/DET, LENT, DIV, and VENT—are sensitive to changes in process stability. Kecik et al. found that six RQA indicators are linked to the stability of the milling process in Inconel 718 [28]. It was shown that in the presence of chatter, the values of DET, RR, LMAX, DIV, LAM, and VMAX significantly increased or decreased. In another study, Ciecieląg, Kecik et al. explored the application of recurrence plots and RQA measures for defect detection in milling processes [29,30]. Their results indicated that DET, TT, VMAX, RPDE, and recurrence times T1 and T2 correlate with the presence of material defects. In a subsequent study, the same researchers demonstrated that up to ten indicators are associated with the occurrence of defects in the milling of composite materials [31]. These include LAM, DET, TRANS, L, ENTR, RPDE, CC, TT, T1, and T2. Ciecieląg also investigated the relationship between technological parameters in the milling of thin-walled composite components and RQA measures [32]. The study revealed that the recurrence indicators L and LAM are linked to feed per revolution and cutting speed, and that LAM also correlates with surface roughness.

The conducted analysis indicates that the use of milling cutters with modified cutting edges—serrated or wavy—affects the dynamics of the milling process due to changes in the actual number of cutting edges in contact with the workpiece. Previous recurrence-based analyses of milling processes have shown that the relationship between recurrence quantification indicators and process dynamics depends on the workpiece material, cutting tool geometry and cutting conditions. Various RQA indicators showed a correlation with the dynamics of the milling process [18,28,29,30,31]. Consequently, the findings from earlier recurrence studies cannot be directly applied to assess the stability of milling processes using tools with serrated cutting edges.

The dynamics of the milling process remain a constant subject of research and analysis. In high-performance milling, tools with interrupted and wavy cutting edges are being used with increasing frequency. As demonstrated by previous research findings, the dynamics of the milling process using tools with discontinuous cutting edges differ from those observed with conventional tools. Therefore, the RQA method was proposed to evaluate the dynamics of the milling process using tools with discontinuous cutting edges. The diagnostic signal was the cutting force. An analysis of the cutting-force components was carried out, and changes in the dynamics of these components were assessed for various cutting parameters. A comparative analysis was performed for tools with different cutting-edge geometries. One component of the cutting force was selected as a representative signal of the milling process dynamics. This signal was subjected to detailed analysis using recurrence methods. Recurrence plots and RQA indicators were analyzed. A group of RQA indicators was identified as suitable for evaluating the dynamics of the milling process when using tools with different cutting-edge geometries. The main novelty of this study lies in the application of the recurrence method to evaluate the dynamics of milling with interrupted and wavy cutting-edge tools, based on the cutting-force signal and the identification of RQA indicators related to milling dynamics.

## 2. Materials and Methods

**Figure 2:**
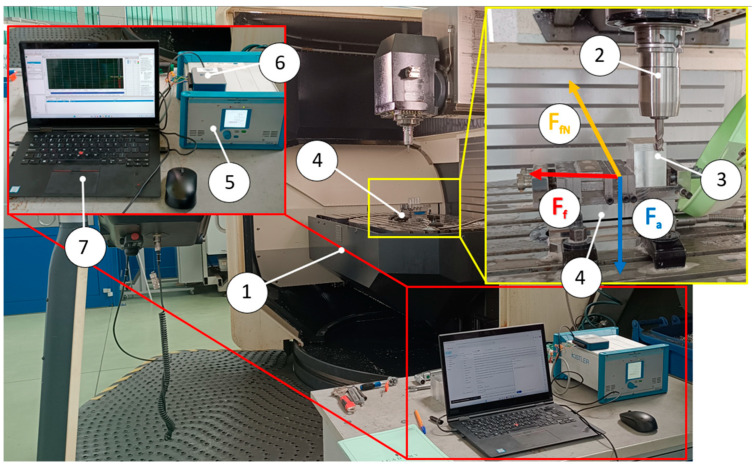
Milling process experimental setup: 1—DMU 100 monoBLOCK machining center; 2—tool holder with cutting tool; 3—test specimen; 4—cutting-force measurement platfor; 5—charge amplifie; 6—A/C data acquisition card, 7—personal computer.

The experimental investigation of the milling process, including the measurement of cutting-force components, was carried out using a DMU 100 monoBLOCK machining center manufactured by DMG (Munich, Germany). This machine is equipped with a spindle power of 19 kW, a torque of 320 Nm, and a maximum spindle speed of 24,000 rpm. The research setup along with its equipment is presented in Figure 2.

In order to record the components of the cutting force, the experimental setup was equipped with a Kistler 9257B (Winterthur, Switzerland) three-axis piezoelectric dynamometer. This system enabled the measurement of the feed force component F_f_, the component normal to the feed direction F_fN_, and the axial force component F_a_. The orientation of the force components was defined in accordance with the workpiece coordinate system (Figure 2). The recorded signals were amplified using a Kistler type 5070 (Winterthur, Switzerland) charge amplifier, then converted into digital form and recorded on a personal computer via a 16-bit NI USB-6003 data acquisition card National Instruments (Austin, TX, USA), with an analog signal sampling frequency of 20 kS/s.

The test specimens were made of AlZn5.5MgCu aluminum alloy. Each specimen had the shape of a cube with dimensions of 50 × 50 × 50 mm. The specimens were mounted on the dynamometer using four clamping screws, which were tightened after each specimen replacement with a torque wrench to a tightening torque of 20 Nm.

The experimental study was conducted according to a two-factor experimental design, in which the influence of one selected machining parameter and the type of cutting tool on the milling process was analyzed. For each of the investigated technological parameters—feed per tooth *f_z_*, cutting speed *v_c_*, and radial infeed *a_e_*—a separate series of experiments was performed. In each series, the value of the investigated parameter was varied while the remaining parameters were kept constant (Table 1).

The second variable factor in the experiment was the type of cutting tool. Three milling cutters with different cutting-edge geometries were used in the study: continuous, serrated, and wavy cutting edges. The tests were conducted using 12 mm diameter end mills manufactured by ENGRAM, specifically designed for high-performance machining of aluminum alloys. The specifications of the milling cutters used in the experiments are presented in Table 2. The tool set was custom-made by Engram, and all tools were uncoated. Based on the manufacturer’s data, the following constant cutting parameters were adopted: cutting speed of 500 m/min, radial depth of cut of 3 mm, and feed per tooth of 0.075 mm/tooth.

Each variation in the investigated machining parameter and cutting tool type was tested independently, allowing for the assessment of the influence of both factors on the analyzed output quantities, including the magnitude and dynamics of the cutting-force components. To ensure consistent and repeatable testing conditions, a surface smoothing pass was performed after each mounting of the test specimen before the test. The obtained cutting-force component signals were subjected to statistical analysis using box plots, followed by recurrence analysis of the signals and evaluation of recurrence quantification indicators.

### 2.1. Box Plots

The box plot, also known as the box–whisker plot, is one of the fundamental methods for graphically presenting the distribution of univariate data. Its primary purpose is to enable quick identification of data dispersion, asymmetry, and the presence of outliers, and to facilitate the comparison of descriptive statistics across different groups.

**Figure 3:**
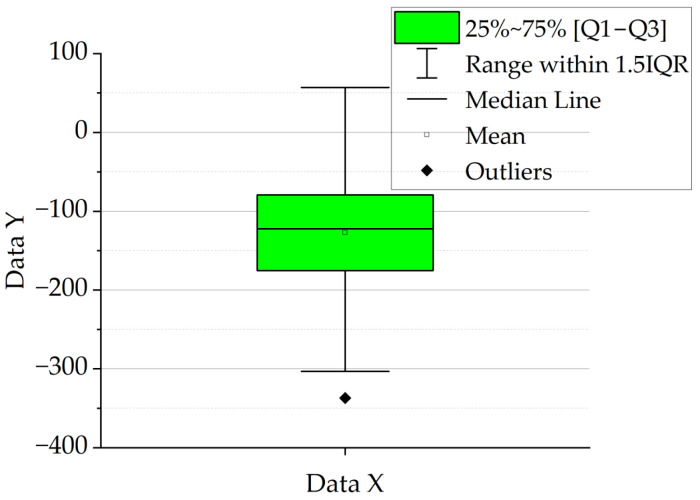
Explanation of the box plot.

The plot consists of a rectangular box representing the interquartile range—from the first quartile (Q1) to the third quartile (Q3)—which contains the central 50% of observations. A line inside the box indicates the median (Q2), while a point inside the box denotes the mean value. The “whiskers” extend beyond the box, typically reaching the minimum and maximum data points within the range of Q1 − 1.5⋅IQR to Q3 + 1.5⋅IQR, where IQR = Q3 − Q1 is the interquartile range. Data points outside this range are considered outliers and are marked as individual points. An example box plot with a detailed explanation is presented in Figure 3.

### 2.2. Recurrence Analysis

The Recurrence Plot (RP), introduced by Eckmann et al. [33], is a square matrix in which each point indicates the recurrence of the system trajectory to a similar state in the phase space. The matrix axes (i, j) correspond to time instances, and recurrence is identified based on parameters: embedding dimension *m*, time delay *τ*, and recurrence threshold *ε* [34].

The parameters for phase space reconstruction, namely the time delay *τ* and embedding dimension *m*, were determined using the Average Mutual Information (AMI) and False Nearest Neighbors (FNN) methods. The time delay *τ* was defined as the first minimum of the average mutual information function:

(1) $$AMI\left(\tau \right)=\Sigma_{i,j}p_{ij}\left(\tau \right)log^{2}\left(p_{ij}\left(\tau \right)/\left(p_{i}p_{j}\right)\right),$$

where $p_{ij}\left(\tau \right)$ is the joint probability of occurrence of the signal value *x*(*i*) and its time-delayed version $x\left(i+\tau \right).$

The embedding dimension *m* was determined using the False Nearest Neighbors (FNN) method, in which a false neighbor satisfies the following criterion:

(2) $$R=\left|x\left(i+m\tau \right)-x\left(NN\left(i\right)+m\tau \right)\right|/|\left|s_{i}^{\left(m\right)}-s_{NN\left(i\right)}^{\left(m\right)}\right||>R_{tol},$$

where $s_{i}^{\left(m\right)}$ denotes the delayed vector, and $R_{tol}$ is the specified tolerance threshold.

Based on the selected parameters, recurrence plots were generated according to the following definition:

(3) $$M_{ij}=\theta \left(\varepsilon -|\left|s_{i}-s_{j}\right||\right),$$

where *θ* is the Heaviside step function, and *s_i_* and *s_j_* are delayed vectors. A recurrence point is denoted as *M_ij_* = 1, while the absence of recurrence is represented by *M_ij_* = 0.

The Recurrence Quantification Analysis (RQA) enabled a quantitative assessment of the signal dynamics during the milling process with cutters of varying geometries.

The Recurrence Plot (RP) method was extended by the Recurrence Quantification Analysis (RQA), proposed by Zbilut and Webber [35], which enables characterization of signal dynamics through the analysis of the density of recurrence points and the structures of diagonal and vertical lines. In the present study, the following indicators were employed.

DET—Determinism. The proportion of recurrence points that form diagonal lines:

(4) $$DET=\frac{\sum_{l=l_{min}}^{N}lP\left(l\right)}{\sum_{i,j=1}^{N}M_{ij}}.$$

L—Average Diagonal Line Length. The mean length of diagonal lines:

(5) $$L=\frac{\sum_{l=l_{min}}^{N}lP\left(l\right)}{\sum_{l=l_{min}}^{N}P\left(l\right)}.$$

Lmax—Longest Diagonal Line. The longest diagonal line excluding the main diagonal:

(6) $$L_{max}=\max\{l_{i}\},\;l_{i}\geq l_{min}.$$

DIV—Divergence. It is the inverse of the longest diagonal line L_max_:

(7) $$DIV=\frac{1}{L_{max}}.$$

ENTR—Entropy. Entropy of the diagonal line length distribution:

(8) $$ENT=-\sum_{l=l_{min}}^{N}p\left(l\right)\log_{2}\left(p\left(l\right)\right).$$

TT—Trapping Time. Average vertical line length:

(9) $$TT=\frac{\sum_{v=v_{min}}^{N}vP\left(v\right)}{\sum_{v=v_{min}}^{N}P\left(v\right)}.$$

Vmax—Max Vertical Line. The longest vertical line:

(10) $$V_{max}=\max\{v_{i}\},\;v_{i}\geq v_{min}.$$

RPDE—Recurrence Period Density Entropy. It characterizes the periodicity of the signal in the context of dynamic systems:

(11) $$RPDE=-\frac{1}{\ln\left(v_{\max}\right)}\sum_{v=1}^{v_{\max}}H\left(v\right)\ln H\left(v\right),$$

where H(v) is the distribution of time shifts *j* and *i* for which $M_{ij}$ = 1.

TRANS—Transition Rate. It quantitatively characterizes the geometric properties of the phase space trajectory:

(12) $$TRANS=\frac{\sum_{i=1}^{N}\sum_{j=1}^{N}\sum_{k=1}^{N}M_{ij}M_{jk}M_{ki}}{\sum_{i=1}^{N}\sum_{j=1}^{N}\sum_{k=1}^{N}M_{ij}M_{ki}}.$$

CC—Clustering Coefficient. It describes the probability that two points are close to each other:

(13) $$CC=\sum_{i=1}^{N}\frac{\sum_{j=1}^{N}\sum_{k=1}^{N}M_{ij}^{m,\varepsilon }M_{jk}^{m,\varepsilon }M_{ki}^{m,\varepsilon }}{\sum_{j=1}^{N}M_{ij}^{m,\varepsilon }}.$$

### 2.3. Linear Discriminant Analysis

Linear Discriminant Analysis (LDA) is a classical statistical and machine learning technique that projects data into a lower-dimensional space by creating discriminant components, which are linear combinations of the original features. The method aims to maximize the separation between predefined classes, such that observations belonging to the same class are as close as possible, while those from different classes are positioned as far apart as possible. From a mathematical perspective, LDA seeks a weight matrix *W* that maximizes the ratio of the between-class scatter matrix *S_B_* to the within-class scatter matrix *S_W_*, according to the following criterion:

(14) $${max}_{W}\;=\frac{det(W^{T}S_{B}W)}{det(W^{T}S_{W}W)},$$

and the solution leads to a generalized eigenvalue problem of the following form:

(15) $$S_{B}w=\lambda S_{w}w,$$

where *λ* denotes the eigenvalue and *w* is the corresponding eigenvector.

The LDA method was applied to assess the suitability of cutting-force components for identifying the dynamic behavior of the milling process using different types of cutters.

## 3. Results and Discussion

### 3.1. The Influence of Machining Parameters and Cutting Edge Type on the Values of Cutting-Force Components

#### 3.1.1. The Influence of Cutting Speed and Cutting Edge Type

**Figure 4:**
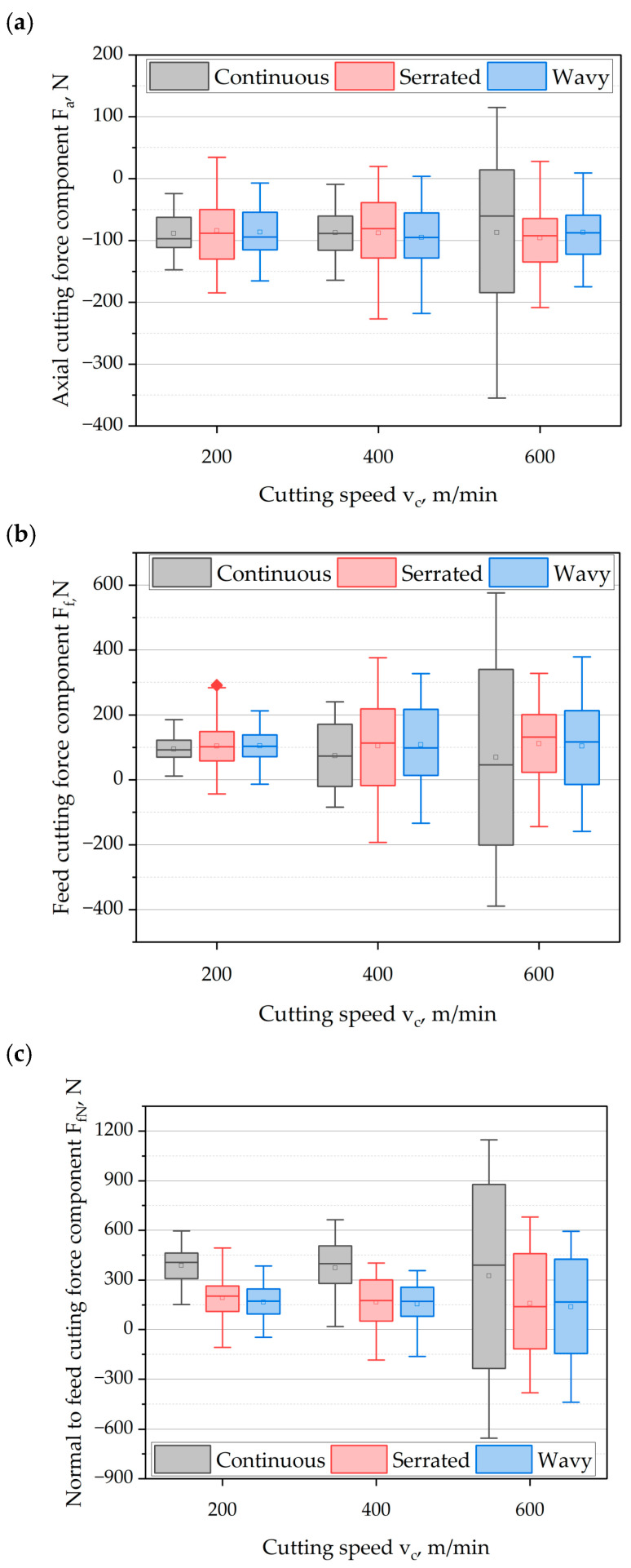
The influence of cutting speed and cutting-edge type on the components of cutting force: (**a**) axial component F_a_; (**b**) feed component F_f_; (**c**) component normal to the feed direction F_fN_.

Figure 4 presents the influence of cutting speed *v_c_* and cutting-edge type on the components of the cutting-force. Analyzing the axial component (Figure 4a) of the cutting-force, it can be observed that the tool generated force values directed opposite to the measurement system, meaning the cutting edge was loading the machine–holder–tool–workpiece (MHTW) system. In contrast, values in the opposite direction represent the response of the entire system resulting from its elasticity.

It can be observed that an increase in cutting speed within the investigated range generally leads to a rise in the axial component F_a_ of the cutting-force for all examined types of cutting edges. A notable case is the cutting speed *v_c_* = 600 m/min, where the maximum value of F_a_ increased by 133% compared to *v_c_* = 200 m/min. In the case of serrated and wavy cutting edges, the values of F_a_ remained at a similar level or were even lower compared to those at lower cutting speeds.

When analyzing maximum values at lower cutting speeds, it can be seen that milling cutters with serrated and wavy edges generated higher maximum axial force values: by 30% and 21%, respectively. On the other hand, considering the mean values of the axial component F_a_ (indicated by the square marker on the plot), all milling tests generally yielded average values around 90 N, and the variations between cutter types and *v_c_* did not exceed 10% of the average.

Analyzing the range of axial force values F_a_, more significant variations were observed for tools with serrated and wavy cutting edges. The only exception was at *v_c_* = 600 m/min, where the continuous edge cutter generated a substantially greater range. This increase is attributed to the destabilization of the machine–holder–tool–workpiece system, resulting from the increased spindle speed *n* of the tool and, consequently, a higher frequency of tool entry into the material. The excitation frequency depends on the number of cutting edges and the spindle speed:

(16) $$f_{e}=z\frac{n}{60},$$

where: *f_e_*—forcing frequency [Hz], *z*—number of cutting edges; *n*—spindle speed [rpm].

For tools with serrated and wavy cutting edges, the force value ranges were observed to be at a similar level as those recorded at lower cutting speeds *v_c_*. It can be assumed that the application of such edge geometries results in a relatively higher number of effective cutting edges, which consequently leads to the generation of significantly higher forcing frequencies. These frequencies may substantially differ from the natural (resonant) frequencies of the MHTW system.

Figure 4b presents the influence of cutting speed and cutting-edge geometry on the maximum, minimum, average values, and the range of variation in the feed force component F_f_. In this case, the tool acts in accordance with the measurement direction of the measurement system, where positive values correspond to the loading of the MHTW system, while the opposite direction represents the elastic response at the moment the force ceases to act. Analyzing the effect of cutting speed *v_c_*, an increase in the maximum values of the feed force component F_f_ was also observed for the tool with a continuous edge. However, for cutting speeds of *v_c_* = 400 m/min and *v_c_* = 600 m/min, the tools with serrated and wavy edge profiles exhibited stabilized maximum force values at approximately 350 N. A notable case is the cutting speed of *v_c_* = 600 m/min, for which the tool with a continuous edge reached a maximum feed force of approximately 570 N, representing an average increase of 43% compared to the other tools.

At lower cutting speeds, an inverse relationship between force magnitude and cutting-edge geometry was observed. Greater maximum force values at lower cutting speeds were recorded for tools with serrated and wavy edges. On average, these were higher than those generated by the continuous-edge tool by:

- For *v_c_* = 200 m/min: 58% (serrated), 14% (wavy),
- For *v_c_* = 400 m/min: 56% (serrated), 35% (wavy).

Considering the average values, it can be observed that at a cutting speed of *v_c_* = 200 m/min, the mean feed force for all cutting-edge types remained at approximately the same level, around 100 N. In contrast, the mean values of the feed force component at *v_c_* = 400 m/min and *v_c_* = 600 m/min were higher for the tools with serrated and wavy edges, on average 44% higher at *v_c_* = 400 m/min and 55% higher at *v_c_* = 600 m/min in comparison to the continuous-edge tool.

Analyzing the variation range of the feed force signal, it can be noted that the tools with serrated and wavy cutting edges generally exhibited a broader range of fluctuation in this component. As observed previously, a specific case is the cutting speed of *v_c_* = 600 m/min, where the tool with a continuous edge demonstrated the largest dispersion in feed force values. This may indicate that the frequency of individual cutting-edge entries into the material was close to the resonance frequency of the MHTW system, which led to increased process dynamics and reduced stability.

For the component of the cutting force normal to the feed direction F_fN_, whose values are presented in Figure 4c, positive values correspond to the interaction of the cutter edges with the workpiece and are aligned with the force sensor’s measurement direction. In contrast, negative values reflect the elastic response of the measurement system during periods when the cutting edge is not in contact with the material. Analyzing the influence of cutting speed on the maximum values of the F_fN_ component, it can be observed that increasing the cutting speed led to an increase in F_fN_ for all tested types of cutting edges. It was also noted that, at each cutting speed *v_c_*, the maximum values of the F_fN_ component were consistently lower for tools with serrated and wavy cutting edges. The percentage changes relative to the continuous-edge tool are as follows:

- *v_c_* = 200 m/min: −17% (serrated), −36% (wavy),
- *v_c_* = 400 m/min: −39% (serrated), −46% (wavy),
- *v_c_* = 600 m/min: −40% (serrated), −48% (wavy).

Considering the average values of the cutting-force F_fN_, it was observed that lower mean values of F_fN_ were consistently obtained for the tools with serrated and wavy cutting edges across the entire tested range of cutting speeds *v_c_*. The relative changes compared to the continuous-edge tool were on average *v_c_* = 200–600 m/min: −53% (serrated), and −57% (wavy). It was also noted that the mean values of the F_fN_ component, depending on cutting speed *v_c_*, remained at a similar level across all cutting-edge types.

Analyzing the range of dispersion of the F_fN_ force component, it was found that the cutter with a continuous cutting edge exhibited a greater spread in values. The largest variation was observed at a cutting speed of *v_c_* = 600 m/min, where the relative difference compared to the serrated and wavy edge tools reached approximately 40%.

#### 3.1.2. The Influence of Feed per Tooth and Cutting Edge Type

**Figure 5:**
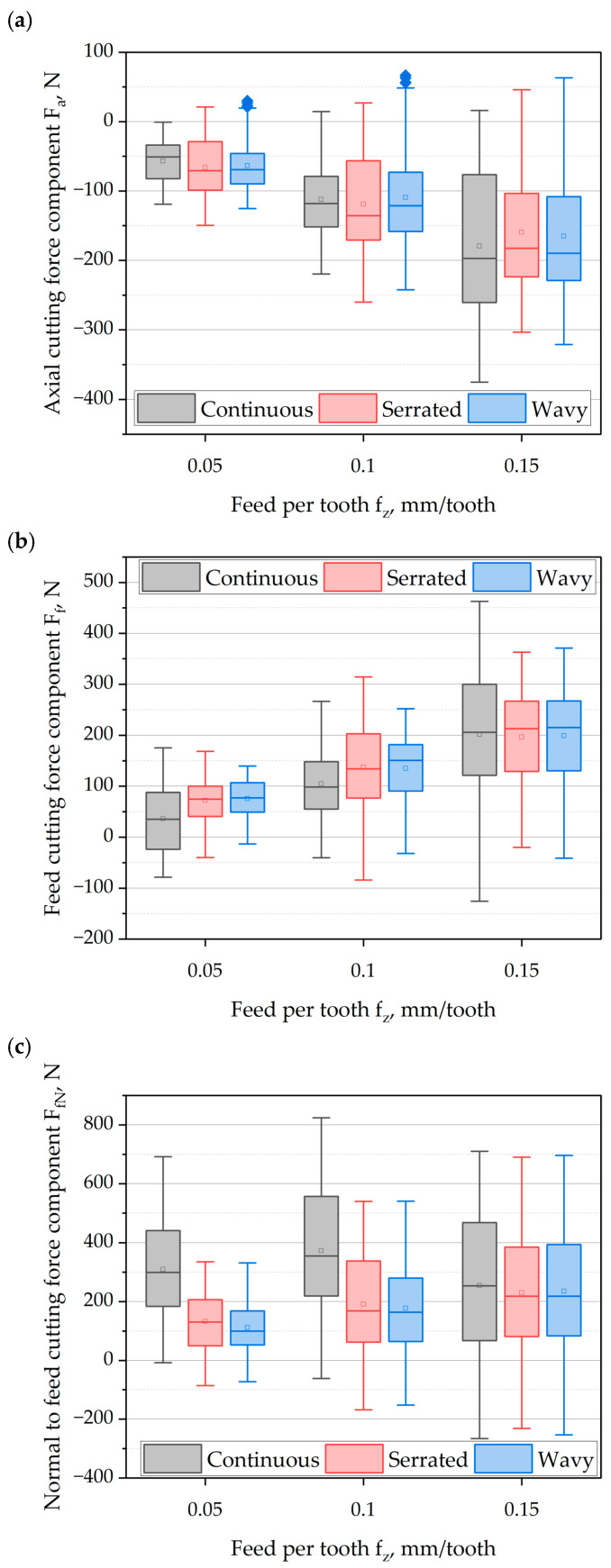
The influence of feed per tooth and cutting-edge type on the components of cutting force: (**a**) axial component F_a_; (**b**) feed component F_f_; (**c**) component normal to the feed direction F_fN_.

Figure 5a shows the changes in the axial component of the cutting force as a function of feed per tooth *f_z_* and the type of cutting edge. Analyzing the absolute values of the maximum axial component F_a_ of the cutting force, it can be observed that with the increase in feed per tooth *f_z_*, the force values increase in a quasi-linear monotonic trend. It can be seen that at lower values of feed per tooth, the maximum values of the F_a_ component of the cutting force occurred for the cutter with an serrated and wavy edge. These values were higher compared to the continuous-edge cutter as follows: *f_z_* = 0.05 mm/tooth (25% serrated, 5% wavy); *f_z_* = 0.1 mm/tooth (18% serrated, 10% wavy). However, in the case of feed per tooth *f_z_* = 0.15 mm/tooth, it was noted that the cutter with a continuous edge generated higher values of the F_a_ component of the cutting force, by 17% compared to cutters with serrated and wavy geometry. It should be noted that the increase in cutting-force components is closely related to the change in feed per tooth *f_z_*, which directly affects the cross-section of the cut layer. The empirical expression of this relationship describes the maximum value of the cross-sectional area of the cut layer *A*:

(17) $$A=bh_{max},$$

(18) $$b=a_{p}\sin\kappa_{r},$$

(19) $$h_{max}=f_{z}\sin\kappa_{r}\sin\varphi ,$$

(20) $$\varphi =\cos^{-1}\left(1-\frac{2a_{e}}{D}\right)$$

where: *A*—cross-sectional area of the cut layer in mm^2^; *b*—width of the cut layer in mm; *h_max_*—maximum thickness of the cut layer in mm^2^; *a_p_*—axial depth of cut in mm; *a_e_*—radial infeed; *f_z_*—feed per tooth in mm/tooth; *D*—tool diameter in mm; *φ*—cutter engagement angle in degrees; *κ_r_*—entering angle—which, for an end mill, equals 90°.

Assessing the average values of the F_a_ component of the cutting-force, it can be observed that the values also increase with the feed per tooth *f_z_* for all the cutters used. Considering the average values depending on the cutter geometry used, it can be seen that at a feed per tooth *f_z_* = 0.05, higher values are generated by the serrated and wavy cutters. For these tools, a relative increase in the force F_a_ was recorded at approximately 17% compared to the cutter with a continuous edge. With further increases in feed per tooth, an opposite trend was observed—cutters with serrated and wavy geometries generated lower axial force values compared to the cutter with a continuous cutting edge. For a feed of *f_z_* = 0.10 mm/tooth, the relative difference was about 4%, while at *f_z_* = 0.15 mm/tooth it reached a negative value of around −9%.

Analyzing the dispersion of F_a_ force values, it can be stated that at feed per tooth *f_z_* = 0.05 and 0.1 mm/tooth, greater force variability is observed for cutters with serrated and wavy cutting edges. Additionally, for both feed values, the cutter with the wavy edge shows the presence of outliers, which may indicate the occurrence of elastic reactions exceeding the typical range. This phenomenon may suggest that the tool with wavy geometry is characterized by higher dynamic behavior, which potentially promotes the formation of self-excited vibrations. Further increase in feed per tooth *f_z_* and the maximum chip thickness *h_max_* causes an increase in the dispersion of the cutting-force component also in the case of the cutter with a continuous cutting edge.

Figure 5b presents the influence of feed per tooth and the type of cutting edge on the values of the feed component F_f_ of the cutting force. A positive value of the force component is the dynamometer’s response to the action of the cutting edge, while lower and negative values are the elastic response of the measuring system to the absence of force. Analyzing the influence of feed per tooth *f_z_* on the maximum value of the feed component of the cutting-force F_f_, it can be observed that for all tested cutters, the maximum values increased monotonically, in a quasi-linear trend, with increasing feed.

An assessment of the influence of the cutting-edge geometry in the analyzed range of feeds showed that the lowest values of force F_f_ in all cases were recorded for the cutter with a wavy cutting edge. The greatest relative reduction compared to the cutter with a continuous edge was observed at *f_z_* = 0.05 and 0.15 mm/tooth—values lower by approximately 20%.

In the case of the cutter with a serrated edge, at a low feed of *f_z_* = 0.05 mm/tooth, the values were comparable to those of the continuous-edge cutter, whereas at *f_z_* = 0.10 mm/tooth, an increase in force of about 18% was recorded. Further increase in feed resulted in more efficient operation of the cutting edge, leading to maximum cutting-force values approximately 20% lower than those of the cutter with a continuous edge.

Considering the average values of the F_f_ component of the cutting force, a monotonic increase in this component as a function of feed per tooth *f_z_* was observed for all analyzed types of cutting tools. It was observed that at feeds of *f_z_* = 0.05 and 0.10 mm/tooth, higher average values of the F_f_ force were obtained for cutters with serrated and wavy cutting edges compared to the cutter with a continuous edge. The relative differences amounted to approximately 101% (for *f_z_* = 0.05 mm/tooth) and 30% (for *f_z_* = 0.10 mm/tooth), respectively. Further increase in feed *f_z_* resulted in a decrease in the average F_f_ force values for cutters with serrated and wavy geometries. A reduction in force of about 30% was recorded compared to the cutter with a continuous cutting edge.

Analyzing the dispersion of values of the F_f_ component of the cutting-force, it can be observed that the cutter with a continuous cutting edge was characterized by greater force variation dynamics. The highest dispersion was recorded at the highest feed per tooth *f_z_*, and it was about 42% greater compared to cutters with serrated and wavy edges.

Figure 5c presents the maximum, minimum, average, and dispersion values of the F_fN_ cutting-force component (perpendicular to the feed direction) as a function of feed per tooth *f_z_* and the type of cutting edge. Analyzing the maximum values of this component, it can be observed that for cutters with serrated and wavy cutting edges, an increase in feed per tooth leads to a quasi-linear, monotonic increase in the F_fN_ force values. In the case of the cutter with a continuous edge, no clear upward trend was observed. The maximum values of this component remained at a similar level regardless of the *f_z_* value, oscillating around an average of approximately 750 N.

Considering the changes in the maximum F_fN_ force values between the tested cutting-edge geometries, it can be seen that the values were consistently lower for cutters with serrated and wavy edges compared to the cutter with a continuous edge. The greatest relative differences occurred at feed per tooth values of *f_z_* = 0.05 and 0.10 mm/tooth. In these cases, the maximum values were lower on average by approximately 52% (for *f_z_* = 0.05 mm/tooth) and 34% (for *f_z_* = 0.10 mm/tooth) compared to the continuous-edge cutter. With further increases in *f_z_*, the maximum force values for cutters with serrated and wavy geometries increased, reaching levels close to those obtained for the continuous-edge cutter. The same conclusions were formulated in Ref [3].

Analyzing the average values of the F_fN_ force, a similar trend of changes in this component can be observed for all analyzed types of cutters as a function of feed per tooth *f_z_*. The most favorable average values were recorded for cutters with serrated and wavy edges—especially for *f_z_* = 0.05 and 0.10 mm/tooth—where these values were clearly lower compared to the continuous-edge cutter. The relative decrease in average values for the serrated and wavy cutters was approximately 70% (for *f_z_* = 0.05 mm/tooth) and 50% (for *f_z_* = 0.10 mm/tooth), respectively, compared to the continuous-edge geometry. For the highest feed of *f_z_* = 0.15 mm/tooth, the average F_fN_ values for all cutter types were at a similar level which confirms the findings reported by Koca and Budak [3].

Analyzing the dispersion of the F_fN_ cutting-force component as a function of feed per tooth *f_z_*, it can be observed that an increase in *f_z_* leads to increased variation dynamics of this component for all analyzed cutting-edge geometries. The most noticeable differences in dispersion were recorded for cutters with serrated and wavy edges. At the lowest feed value (*f_z_* = 0.05 mm/tooth), the F_fN_ force dispersion was approximately 40% lower compared to the cutter with a continuous cutting edge. For the feed of *f_z_* = 0.15 mm/tooth, the spread values for all tool geometries were already at a similar level. The similar cutting-force values observed for tools with continuous and discontinuous cutting edges at the highest feed per tooth values result from the reduced segmentation effect of the machined layer in the case of serrated and wavy tools. As the chip thickness increases, the cutting edge penetrates deeper into the material, and the influence of the cutting-edge profile becomes progressively less significant [3,12,16].

#### 3.1.3. The Influence of Radial Infeed and Cutting Edge Type

**Figure 6:**
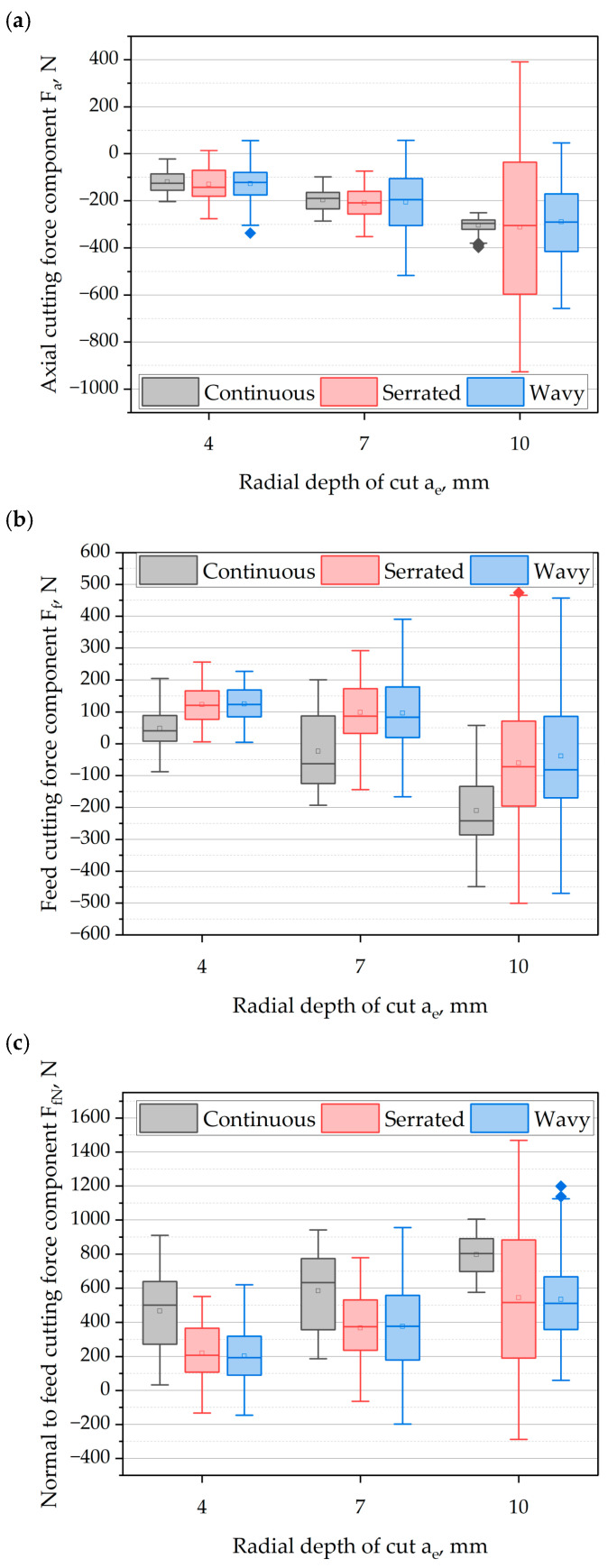
The influence of radial infeed *a_e_* and cutting-edge type on the components of cutting force: (**a**) axial component F_a_; (**b**) feed component F_f_; (**c**) component normal to the feed direction F_fN_.

Figure 6a presents the values of the axial component F_a_ of the cutting force as a function of the radial infeed *a_e_* and the type of cutting edge. The analysis of the absolute maximum values indicates that increasing the *a_e_* causes a monotonic, quasi-linear increase in the F_a_ component for each of the tested tool types. Comparing the influence of the cutting-edge geometry, it can be observed that in all cases, higher maximum F_a_ force values were recorded for cutters with serrated and wavy edges compared to the cutter with a continuous edge. The greatest differences occurred at the highest cutting radial infeed *a_e_*, where the cutter with the serrated edge generated an F_a_ component value about 130% higher, and the wavy-edge cutter about 68% higher, compared to the continuous-edge cutter.

Analyzing the average values of the axial component F_a_ of the cutting force, it can be observed that this component increased with feed per tooth *f_z_* for all analyzed cutting-edge geometries. However, no significant influence of the cutting-edge type on the average F_a_ values was recorded—the differences between the tested cutters were slight. For each cutting radial infeed *a_e_*, the average values remained at a similar level regardless of edge geometry.

Assessing the dispersion of the axial component F_a_ of the cutting force, it can be seen that increasing the cutting radial infeed *a_e_* leads to a clear stabilization of the cutting process in the case of the cutter with a continuous cutting edge. This is confirmed by the presence of a narrow box and short whiskers on the plot. This phenomenon can be explained by greater tool engagement and simultaneous involvement of a larger number of cutting edges, which, with a large helix angle of the cutting edge, results in the stress stiffening effect (stiffening of the system due to internal stresses). In contrast, for cutters with serrated and wavy edges, a significantly greater dispersion in F_a_ force values was observed. This may indicate that the segmented edge geometry generates entry frequencies of the tool close to the resonant frequencies of the MHTW system, leading to increased dynamic instability of the process.

Figure 6b presents the influence of cutting radial infeed *a_e_* and cutting-edge type on the maximum, average, and dispersion values of the feed component F_f_ of the cutting force. Analyzing the maximum values of this component, it can be noted that as the cutting radial infeed *a_e_* increases, the direction of the force action changes from positive to negative. This effect results from the increasing overlap of cutting edges remaining in contact with the workpiece. As a result, at a small cutting radial infeed *a_e_*, the F_f_ component acts more in the direction of the workpiece, whereas at higher *a_e_* it acts more in the direction of the cutting tool. This is particularly visible in the average values of the F_f_ component of the cutting force. It can be observed that increasing the *a_e_* contributes to the growth of the F_f_ cutting-force component. For all analyzed cutting-edge types, this increase is nonlinear.

Assessing the dispersion of the feed component F_f_ of the cutting force, it can be observed that in most cases, greater variation occurred for cutters with serrated and wavy cutting edges. This effect is particularly visible for a cutting infeed of *a_e_* = 10 mm, where the force F_f_ dispersion for the cutter with an serrated edge was about 92% greater, and for the wavy edge about 82% greater, compared to the cutter with a continuous edge.

Figure 6c presents the influence of cutting radial infeed *a_e_* and cutting-edge type on the values of the F_fN_ force component, perpendicular to the feed direction. The analysis of maximum values indicates that increasing the cutting infeed *a_e_* causes a quasi-linear increase in maximum F_fN_ values for all analyzed edge geometries. Considering the influence of cutting-edge type, it can be seen that only at an infeed of *a_e_* = 4 mm were lower maximum values of F_fN_ obtained for the serrated-edge cutter (about 40%) and the wavy-edge cutter (about 30%) compared to the continuous-edge cutter. With further increases in cutting infeed, the opposite trend was noted—at *a_e_* = 10 mm, the serrated cutter generated a maximum F_fN_ value about 40% higher, and the wavy cutter was about 13% higher than the continuous-edge cutter.

Analyzing the average values of the F_fN_ component, a monotonic, linear increase in this component was observed with increasing cutting width for each of the tested cutters. Additionally, regardless of *a_e_* value, the average F_fN_ force values were lower for cutters with serrated and wavy edges compared to the continuous-edge cutter. The relative decrease in average values was about 50% for *a_e_* = 4 mm and about 33% for *a_e_* = 7 and 10 mm. In turn, the analysis of dispersion indicates that increasing the cutting infeed leads to a significant rise in variability of the F_fN_ component in the case of cutters with serrated and wavy edges. For *a_e_* = 10 mm, the serrated-edge cutter generated a dispersion about 300% greater, and the wavy-edge cutter about 160% greater compared to the continuous cutter.

Similar conclusions were reached by Burek et al., who investigated the influence of cutting-edge type on the milling process over a wide range of cutting parameters [15,36]. They demonstrated that, as in the present study, the F_fN_ component for serrated and wavy-edge tools was lower than for the continuous-edge tool, while the F_f_ component showed an opposite trend in most cases.

### 3.2. The Influence of Technological Parameters and Cutting Edge Geometry on Recurrence Plots (RP)

The recurrence analysis method was used to analyze the dynamics of systems and processes. Therefore, an attempt was made to analyze the milling process using cutters with different cutting-edge geometries using means of the recurrence method. For this purpose, a Python (version, 3.13) script was developed, which enabled the calculation of RQA indicators and RP plots. Table 3, Table 4 and Table 5 present the calculated values of RQA indicators as a function of technological parameters and cutting-edge type.

Due to the large amount of data from the three components of the cutting force, the LDA method was applied to determine a single force component that provides the highest effectiveness in identifying the type of cutter. The aim was to separate the cutter with a continuous cutting-edge profile from those with wavy and serrated profiles.

**Figure 7:**
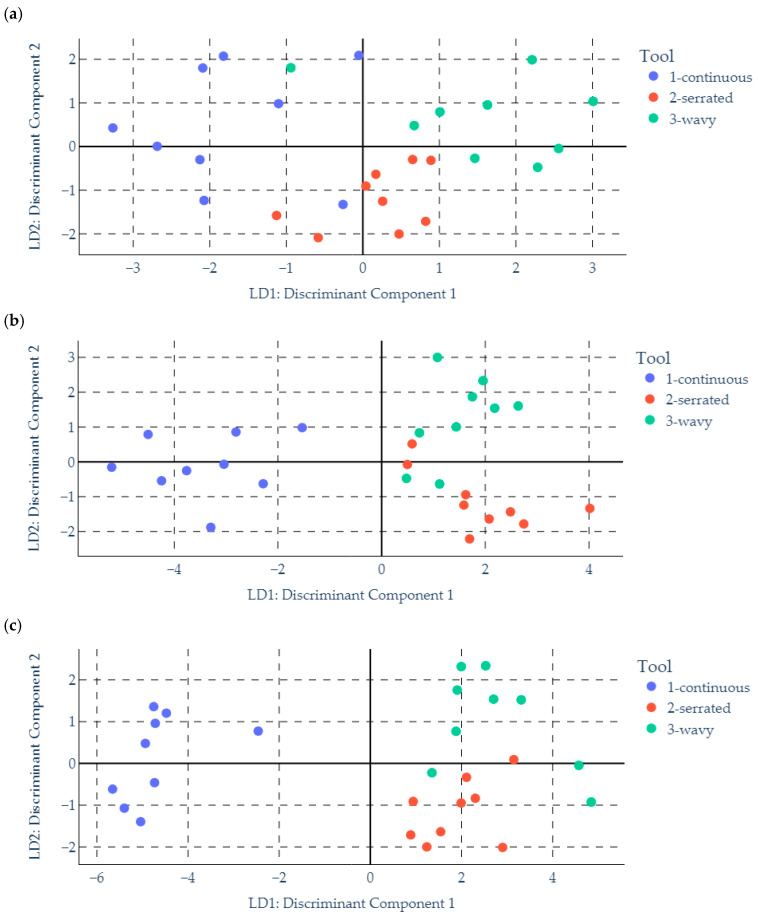
The LDA analysis of cutting-force components: (**a**) F_f_; (**b**) F_fN_; (**c**) F_a_.

Figure 7 presents the results of the LDA analysis for each force component separately. The analysis shows that the force components F_fN_ and F_a_ allow for a clear separation of the data sets of the continuous-edge tool from those with wavy and serrated edges. However, greater effectiveness in identifying the cutting-edge type—measured by the distance between the two data sets—is demonstrated by the F_a_ component. Therefore, only the signal from the F_a_ force component was used for further recurrence analysis.

Analyzing the obtained images of the milling process dynamics using recurrence methods, it can be observed that changes in cutting speed and cutting-edge type significantly affect the resulting recurrence plots (Table 6). Thus, it can be concluded that both the tool and the cutting speed have a clear impact on the dynamics of the MHTW system.

Analyzing the plots for the cutting speed *v_c_* = 200 m/min, it can be noted that for the tool with a continuous profile, distinct, regular, thick, long diagonal bands parallel to the main central diagonal of the RP plot were observed, indicating high determinism and process stability (Table 6). In the case of the tool with an serrated profile, the structure of the plot was less coherent—the presence of broken diagonals and local blocks indicated moderate stability and cyclic dynamic fluctuations of the signal. For the tool with a wavy profile, the RP plot was highly dispersed, suggesting a chaotic signal character and a low level of process dynamic regularity.

At a cutting speed of *v_c_* = 400 m/min, different dynamic responses of the milling process were observed depending on the tool type. The tool with the continuous profile still showed high regularity and determinism. For the serrated profile, an increase in RP irregularity was observed—the structure became more granular, which may indicate increased chaos in the milling process. Meanwhile, for the wavy profile, quasi-periodic patterns appeared, indicating local structures and phase transitions in the dynamics; however, overall stability remained low.

A further increase in cutting speed led to an improvement in the dynamics of the milling process for the serrated and wavy tools. For the serrated profile, the RP plot became clearly more organized—long diagonals appeared, which may indicate a synchronization effect or the stabilization of machining conditions at high speed. Also, the tool with a wavy profile, which previously showed the highest level of irregularity, began to generate ordered RP structures, which may suggest the occurrence of resonance between the tool’s characteristics and the frequency of interaction with the material. Meanwhile, the tool with the continuous profile maintained very high stability and determinism despite a significant increase in process energy, which correlates with the large dispersion shown in Figure 4a.

Table 7 presents the RP plots for variations in feed per tooth *fz* and cutting-edge type. Analyzing the images presented in Table 7, it can be observed that changes in feed per tooth affect the dynamics of the milling process for all types of cutting-edge geometry. Considering the lowest feed per tooth, the obtained RP plots were characterized by high regularity and determinism, especially for the cutter with a continuous profile. The diagonals were distinct and long, and the number of scattered points was minimal. For cutters with wavy and serrated edges, signals also showed repeatability patterns along the diagonals, but the diagonals exhibited greater dispersion of recurrence points. In the case of the serrated-edge tool, small local breaks in the diagonals appeared, but the process remained very well-ordered. A more periodic signal behavior was observed for the wavy-edge tool. In this case, greater dispersion of recurrence points along the diagonals was noted, which corresponds to outlier values in Figure 5a (this may indicate greater process dynamics).

With an increase in feed per tooth to 0.10 mm/tooth, a slight increase in granularity and local disturbances in the RP structure were observed in all cases. The diagonals remained the main element of the plots, but they became more serrated and were complemented by short segments and small scattered points. Despite the appearance of these disturbances, the signals still exhibited high stability and predictability of process dynamics, regardless of the edge profile.

The highest feed per tooth *fz* resulted in a further increase in the irregularity of the cutting-force signal, which was reflected in the RP plots by a greater number of breaks and local disruptions along the diagonals. A particularly noticeable increase in “granularity” was visible for the tool with the wavy profile, where the highest number of scattered points and irregular short structures appeared. Nevertheless, the main periodic pattern was still present for all tools, with the tools featuring a continuous profile showing the highest resistance to disturbances.

Analyzing the RP plots presented in Table 8, it can be observed that the radial depth of cut *a_e_* significantly influences the dynamics of the milling process. This is likely due to the change in tool engagement angle as the tool’s radial involvement with the workpiece increases.

At the lowest analyzed *a_e_*, the RP plots show clear periodicity and high regularity for all types of tools. The tool with a continuous profile is characterized by the longest and most compact diagonals, confirming its high stability and deterministic signal nature. For the serrated and wavy tools, noticeable “granularity” and local disturbances appear, and the wavy tool in particular already shows initial signs of irregularity and dispersion in the RP structures.

With an increase in milling infeed *a_e_* to 7 mm, the RP structure clearly degrades. The tool with a continuous profile still maintains periodic patterns, but the number of scattered points and local diagonal breaks increases significantly. For the serrated tool, the diagonals are short and perforated, and the background of the plot becomes dominated by irregular blocky clusters. The milling process is most prone to destabilization when using the wavy tool—the RP structure becomes almost completely dispersed, and periodicity nearly disappears, indicating a clearly chaotic signal character.

The largest milling infeed *a_e_* leads to further intensification of signal irregularity and chaos, especially for tools with serrated and wavy profiles. Long diagonals in the RP almost completely vanish, and the plot consists of short segments and scattered points, indicating a transition to a highly unstable and irregular state. Only the continuous tool retains some residual periodicity, although even here a strong increase in local disturbances is noticeable.

The image-based analysis allows only a subjective evaluation of the influence of a given parameter and cutting edge type on dynamic changes in the MHTW system. Therefore, in the next part of the study, an analysis of selected key recurrence indicators was conducted, which show significant changes depending on the tool type and technological parameters used.

In practice, a large number of different recurrence indicators can be defined. However, their usefulness for assessing a specific process or phenomenon varies. Therefore, a detailed analysis should be performed only for those indicators that show a relationship with the studied process or phenomenon. For this reason, the following method was adopted to eliminate irrelevant indicators. Absolute changes in the values of all indicators were calculated for the cutter with a continuous profile and for cutters with serrated and wavy profiles. A significance threshold of 10% was adopted. This means that only those indicators that showed average absolute changes greater than 10% between the continuous cutter and the wavy and serrated cutters were analyzed. Based on this criterion, it was found that only six indicators are sensitive to changes in the dynamics of the analyzed milling process. Therefore, only these six indicators were subjected to further analysis.

### 3.3. The Influence of Technological Parameters and Cutting Edge Geometry on RQA Indicators

**Figure 8:**
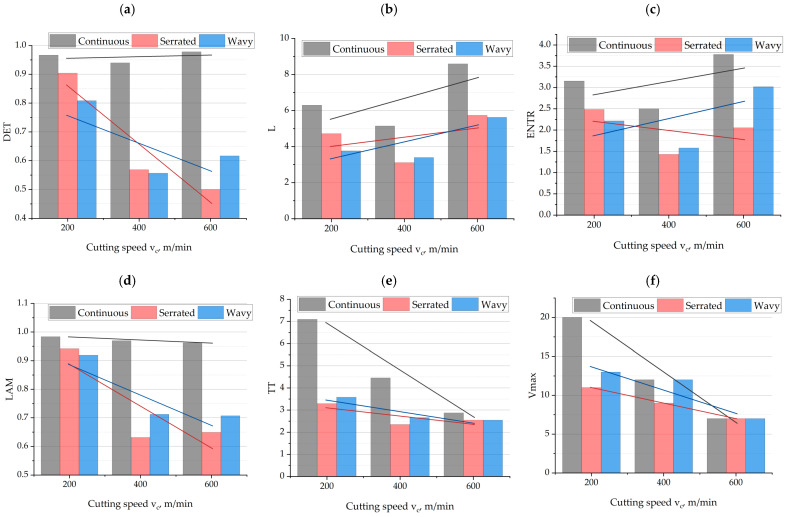
The influence of cutting speed and cutting-edge type on RQA indicators: (**a**) DET; (**b**) L; (**c**) ENTR; (**d**) LAM; (**e**) TT; (**f**) Vmax.

Figure 8 presents the results of the recurrence indicators as a function of cutting speed *v_c_* and cutting-edge type. The analysis of the determinism indicator (DET) in Figure 8a reveals significant differences in the nature of the cutting process dynamics depending on the applied tool geometry and cutting speed. The cutter with a continuous edge shows the highest DET values at all analyzed cutting speeds (200, 400, 600 m/min), indicating a more ordered, deterministic course of the process. For serrated and wavy geometries, a clear decrease in DET was observed with increasing *v_c_*, particularly for the serrated cutter, for which DET decreases from approx. 0.9 at 200 m/min to approx. 0.5 at 600 m/min. This trend indicates an increase in the chaotic and irregular nature of the cutting-force signal as the cutting speed increases. In the case of the wavy geometry, the decrease in DET is also noticeable but slightly milder. The obtained results suggest that the cutting-edge geometry has a key influence on the stability of the machining process, and the use of a continuous cutting edge promotes a more deterministic character of the process regardless of *v_c_* value. The high sensitivity of the DET indicator to changes in process dynamics was confirmed in ref [30]. DET was identified as one of the most suitable parameters for detecting defects in the process, i.e., dynamic changes.

The average diagonal line length indicator L (Figure 8b), which reflects the average duration of states with similar trajectories in phase space, shows an increasing trend for all tool geometries as cutting speed *v_c_* increases. The highest L values were obtained for the continuous edge, reaching above eight at *v_c_* = 600 m/min, confirming a high degree of regularity and predictability in the process course. In the case of serrated and wavy edges, L values increase much more slowly, reaching similar levels of about 5.7 at the highest speed. For low cutting speeds (200 m/min), the differences between the geometries are more pronounced—the continuous cutter reaches an L value of six, while the serrated and wavy geometries do not exceed five. The observed increase in L with increasing *v_c_* suggests that regardless of geometry, the cutting process becomes more dynamically ordered at higher speeds; however, a clear advantage in terms of dynamic stability is maintained by the cutter with a continuous edge.

The diagonal structure entropy indicator ENTR (Figure 8c), which describes the complexity and irregularity of the cutting process, reveals significant differences between the analyzed tool geometries. The highest ENTR values were recorded for the continuous-edge tool, with a noticeable upward trend—from approximately 3.2 at *v_c_* = 200 m/min to over 3.6 at *v_c_* = 600 m/min. This indicates that despite the high level of determinism and regularity (high DET and L), the cutting process using a continuous-edge tool is also characterized by high complexity, which may result from multistage interactions between the tool and the material. For the wavy geometry, entropy also increases with speed, reaching around three at *v_c_* = 600 m/min, suggesting a gradual increase in the complexity of process dynamics. In contrast, for the serrated-edge tool, ENTR decreases, indicating simplification and a possible transition of the process toward more regular but less complex trajectories. This variation in trends suggests that tool geometry affects not only the stability and predictability of the process (DET, L) but also its dynamic complexity, which should be considered when selecting machining strategies.

The laminarity indicator LAM (Figure 8d), which measures the presence of vertical structures in the RP plot and reflects the occurrence of “frozen” or less dynamic states in the signal, shows the highest values for the continuous-edge tool regardless of cutting speed. LAM values for this geometry remain close to the maximum value of 1, indicating a highly ordered and stable process. For serrated and wavy edges, a clear decrease in LAM is observed as *v_c_* increases, suggesting deepening disturbances in the cutting process dynamics, likely due to the discontinuity of tool–material contact. For the serrated-edge tool, LAM drops from approximately 0.94 at *v_c_* = 200 m/min to around 0.6 at *v_c_* = 600 m/min, indicating significant instability and increased chaotic behavior of the process. A similar but slightly milder trend is observed for the wavy geometry. These results confirm that a continuous-edge geometry promotes high regularity and smoothness in machining.

The trapping time indicator TT (Figure 8e), which measures the average time the system spends in stationary states, shows a strong dependence on tool geometry and cutting speed. For the continuous-edge tool, a clear decrease in TT is observed with increasing *v_c_*—from values above seven at 200 m/min to around three at 600 m/min. This trend suggests shorter periods of relative system stabilization as cutting intensity increases. For the serrated and wavy-edge tools, TT values are significantly lower and less variable—remaining in the range of 2.5–3.5 regardless of speed. This behavior may indicate more frequent dynamic transitions and higher momentary variability in the cutting-force signal, characteristic of tools with discontinuous edges. Overall, the continuous geometry favors longer periods of steady-state behavior, which may be beneficial in terms of machining quality and process predictability, especially at lower cutting speeds. The results obtained from the analysis of the TT indicator confirm the conclusions reported in Ref [30].

The Vmax indicator (Figure 8f), interpreted as a measure of the longest period the system remains in a trapped (non-dynamic) state, shows a clear decline with increasing cutting speed *v_c_* for all tool geometries. The highest Vmax values occur at low speed (200 m/min) and with the continuous geometry—reaching a level of 20, indicating very long episodes of stable, low-variability system states. As *v_c_* increases, these values decrease, reaching 7 for the continuous-edge tool at 600 m/min. A similar trend is observed for the serrated and wavy-edge tools, though with lower initial and final values (from ~13 and ~14 to ~7, respectively). This decrease in Vmax indicates an increase in system dynamics and a reduction in the duration of stability periods in the cutting-force signal at higher speeds. These results further emphasize that a continuous geometry promotes more stable and predictable dynamic states at lower machining parameters, whereas discontinuous geometries generate a more variable process course.

**Figure 9:**
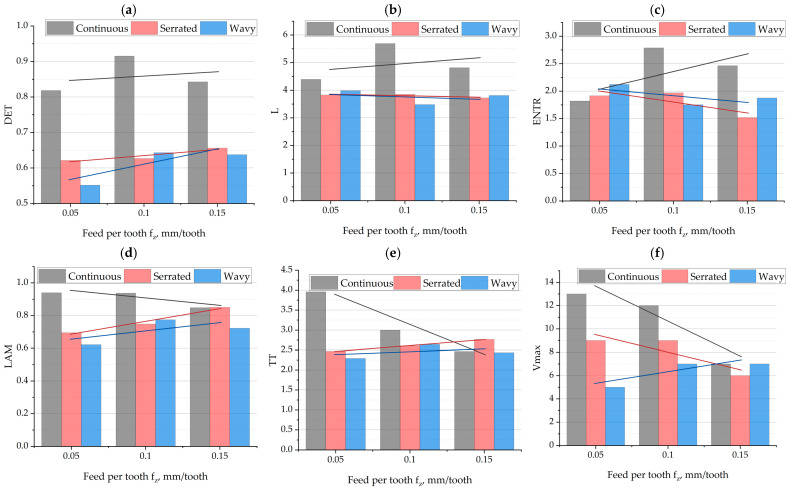
The influence of feed per tooth and cutting-edge type on RQA indicators: (**a**) DET; (**b**) L; (**c**) ENTR; (**d**) LAM; (**e**) TT; (**f**) Vmax.

Figure 9 presents the results of the influence of feed per tooth *f_z_* and cutting-edge type on the recurrence indicators. A similar analysis was conducted to determine the parameters that, under the given experimental conditions, exhibited an average absolute value difference greater than 10% compared to the continuous-edge cutter.

The analysis of the determinism indicator DET (Figure 9a) as a function of feed per tooth *f_z_* reveals a varied response of the dynamic system depending on the tool geometry. The cutter with a continuous edge exhibits the highest DET values across all analyzed feed values, reaching a maximum at *f_z_* = 0.1 mm/tooth (DET ≈ 0.92), indicating an exceptionally stable and orderly cutting process under this setting. For serrated and wavy geometries, DET values are noticeably lower—oscillating in the range of 0.55–0.65—but show a mild upward trend with increasing *f_z_*. This may suggest that higher feed stabilizes the process for less regular edge geometries, reducing the influence of local disturbances and irregular tool–material interactions. As *f_z_* increases, the specific cutting resistance of the material decreases, and thus the tool load also decreases. Nevertheless, the continuous geometry remains by far the most favorable in maintaining a deterministic dynamic behavior of the system over the full range of feed values analyzed.

The average diagonal line length indicator L (Figure 9b), shows moderate variation depending on the tool geometry and *f_z_* value. The continuous-edge cutter reaches the highest L values, particularly at *f_z_* = 0.1 mm/tooth, where it exceeds five, indicating a more stable and predictable process. For serrated and wavy tools, the L indicator remains at a similar level throughout the analyzed feed range, around 3.8–4.0. The lack of a clear increasing or decreasing trend for these geometries suggests that the average duration of dynamically repeating episodes is relatively independent of *f_z_*, whereas the continuous geometry provides better conditions for longer ordered states. These conclusions correlate with the earlier DET analysis.

The entropy indicator ENTR (Figure 9c), shows significant differences between tool geometries and a moderate dependence on *f_z_*. For the continuous-edge tool, ENTR reaches the highest values, peaking at around 2.7 at *f_z_* = 0.1 mm/tooth, indicating high system complexity—despite its simultaneously ordered nature (high DET and L). For serrated and wavy tools, entropy remains lower and more uniform (1.8–2.0), showing a slight downward trend with increasing *f_z_*. This may indicate that for discontinuous tools, higher *f_z_* values lead to simplification of dynamic trajectories and lower process complexity. Overall, the continuous geometry favors both orderly and complex dynamics, which may be beneficial for advanced control and prediction of MHTW system behavior.

The laminarity indicator LAM (Figure 9d), shows interesting variation among tool geometries. For the continuous-edge tool, LAM remains high (above 0.85) and only slightly decreases with increasing *f_z_*, confirming the relatively stable nature of the process regardless of feed intensity. An opposite trend is observed for serrated and wavy tools—here, LAM increases with increasing *f_z_*, reaching values close to those of the continuous-edge tool at the highest feed. This effect may indicate that higher *f_z_* values reduce the influence of irregularities caused by discontinuous geometry, leading to more uniform periods of tool–material contact. It is worth noting that at the lowest *f_z_*, serrated and wavy geometries show distinctly lower laminarity, suggesting a higher level of disturbances and transitional dynamic states in the signal.

The average trapping time indicator TT (Figure 9e), shows different behavior depending on tool geometry. The continuous-edge cutter exhibits the highest TT value at the lowest feed per tooth (over 4), with a clear downward trend as *f_z_* increases. This indicates shortening of relative system stabilization periods under more intense cutting conditions. In contrast, serrated and wavy tools have lower but stable TT values (~2.5–2.8), showing a slight increase with increasing *f_z_*. This may suggest that increasing *f_z_* improves contact continuity for discontinuous tools, resulting in slightly longer and more regular episodes in the cutting-force signal. Nevertheless, the continuous-edge tool dominates in terms of stable state duration only at low *f_z_*, indicating greater sensitivity of this geometry to changing cutting intensity.

The Vmax indicator (Figure 9f), shows a clear decline with increasing *f_z_* for the continuous-edge tool—from around 13 at *f_z_* = 0.05 mm/tooth to about 7 at *f_z_* = 0.15 mm/tooth. This means that at low feeds, the system remains in stable dynamic states for longer, which are shortened as the process becomes more intense. For serrated and wavy tools, lower initial values and slower decline (or even a slight increase in the case of the wavy tool) were observed, which may indicate greater resistance to dynamic changes with increasing *f_z_*, though at the cost of overall lower stability. These results are consistent with earlier observations of the TT and LAM indicators.

**Figure 10:**
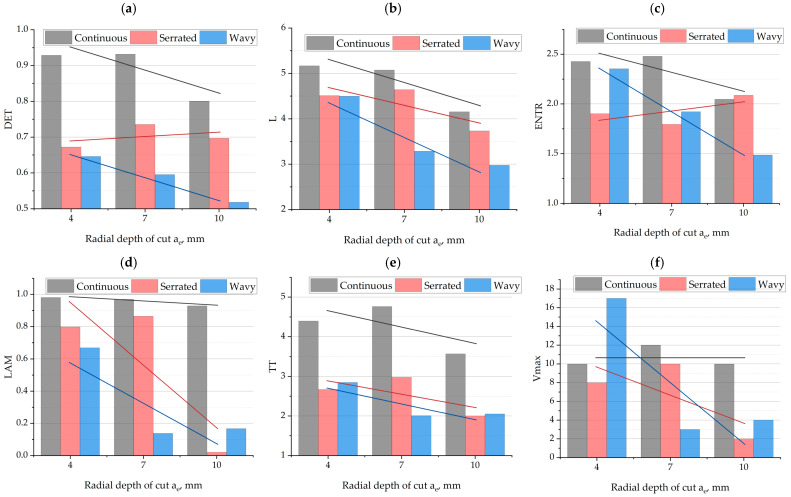
The influence of radial infeed and cutting-edge type on RQA indicators: (**a**) DET; (**b**) L; (**c**) ENTR; (**d**) LAM; (**e**) TT; (**f**) Vmax.

Figure 10 presents the results of the influence of radial infeed *a_e_* and cutting-edge type on the previously selected recurrence indicators. The determinism indicator DET (Figure 10a), shows a strong dependence on both tool geometry and radial infeed *a_e_*. For the continuous-edge cutter, DET reaches the highest values in all tested conditions, exceeding 0.9 at *a_e_* = 4 and 7 mm. The relative decrease in DET at *a_e_* = 10 mm (to ~0.8) suggests a weakening of the deterministic nature of the process under heavier cutting conditions. For the serrated geometry, a slight increase in DET is observed from 4 to 7 mm followed by stabilization, which may indicate that moderate cutting depths mitigate the irregularity effect of the tool. In contrast, for the wavy geometry, DET clearly decreases with increasing *a_e_*—from around 0.65 to about 0.5—indicating an increase in chaotic and irregular structures in process dynamics. The data confirm that the continuous geometry provides the highest level of order in recurrence trajectories, even under intensified machining conditions. The usefulness of the DET indicator for detecting defects and loss of stability in the milling process was also demonstrated in the works of Kecik, Rusinek, and Ciecielag [18,29,31]. This confirms that DET is highly sensitive to changes in the dynamic behavior of the machining process.

The L indicator (Figure 10b), shows a clear downward trend for all edge geometries as the cutting radial infeed *a_e_* increases. The highest L values were obtained for the continuous geometry, with a value of about five at *a_e_* = 4 mm decreasing to around 4 four at *a_e_* = 10 mm. For serrated and wavy edges, L values were lower and also decreased with increasing *a_e_*, reaching approximately 3.5 (serrated) and 3.0 (wavy) at the highest depth. The overall decrease in the L indicator indicates shorter durations of ordered dynamic episodes in the cutting-force signal, which can be interpreted as increasing process instability with larger material engagement. The clear advantage of the continuous edge in trajectory length confirms its ability to sustain more stable and regular dynamic states.

The entropy indicator ENTR (Figure 10c), shows distinctly different trends for various edge geometries as a function of radial infeed *a_e_*. For the continuous geometry, a slight downward trend is observed—from around 2.4 at *a_e_* = 4 mm to about 2.1 at *a_e_* = 10 mm—suggesting a decrease in process complexity under more aggressive machining parameters. A more significant drop in ENTR is observed for the wavy geometry, where entropy decreases from ~2.3 to ~1.4, indicating simplification of dynamics and the dominance of repetitive trajectories. In contrast, the serrated geometry shows a mild upward trend in entropy with increasing *a_e_*, which may signal increasing signal irregularity due to more frequent disturbances resulting from discontinuous tool–material contact. The diverse behavior of entropy reflects the nonlinear influence of radial infeed *a_e_* on the structure of dynamic trajectories and highlights the role of edge geometry in shaping this complexity.

The laminarity indicator LAM (Figure 10d), reveals significant differences between edge geometries as a function of radial infeed *a_e_*. For the continuous-edge tool, LAM remains very high (above 0.9) across all analyzed *a_e_* values, confirming a very stable and orderly process regardless of machining intensity. For serrated and wavy geometries, a clear decrease in LAM is observed with increasing *a_e_*—particularly significant for the wavy edge, where LAM drops from ~0.65 to only ~0.15. This indicates increasing irregularity and unstable dynamic transitions under greater radial tool engagement. A similarly strong drop in LAM is observed for the serrated geometry, underscoring the system’s sensitivity to radial infeed *a_e_* in the case of tools with discontinuous cutting-edge contact. These results clearly indicate the superiority of continuous geometry in maintaining laminar process characteristics under increasing load.

The TT indicator (Figure 10e), shows strong dependence on edge geometry and cutting width. For the continuous geometry, TT values are the highest in all cases, peaking above 4.5 at *a_e_* = 7 mm and remaining above 3 at other values. For serrated and wavy geometries, TT values are distinctly lower (2–3) and exhibit a downward trend with increasing *a_e_*. This means that only the continuous geometry enables longer phases of relative signal stability at moderate cutting depths, while discontinuous geometries become increasingly unstable as tool load increases. The consistency of this observation with the LAM and DET trends confirms the dominant influence of edge geometry on the temporal structure of process dynamics. The usefulness of the LAM and DET indicators for detecting changes in milling process dynamics was confirmed in studies [18,29]. These indicators were successfully used to identify transitions in the milling process state from stable to unstable.

The Vmax indicator (Figure 10f) shows a varied system response depending on edge geometry and cutting radial infeed *a_e_*. For the continuous geometry, Vmax values remain high and relatively stable (~11–13) regardless of cutting radial infeed *a_e_*, suggesting the persistence of long episodes of relatively unchanging dynamics even under more intense machining conditions. For the non-continuous geometry, a clear downward trend is observed—from ~10 to only ~2.5—indicating a shortening of stable states under increased tool–material contact. An even stronger drop is observed for the wavy geometry—from a very high initial value (~17) to ~4—indicating a significant increase in system instability. Despite its initially high Vmax, the wavy geometry proves to be the least resistant to increasing *ae*, while the continuous geometry remains the most stable.

## 4. Conclusions

Based on the conducted experimental investigations, it can be conclusively stated that the geometry of the cutting edge significantly influences both the magnitude of the cutting-force components and the dynamic behavior of the milling process for AlZn5.5MgCu aluminum alloy. RQA constitutes a powerful methodology for assessing milling process dynamics and offers diagnostic potential for predicting process stability depending on tool geometry and machining parameters. Based on the conducted research and analyses, the following conclusions have been formulated:

- Tools with serrated and wavy cutting edges generated substantially lower values of the normal cutting-force component F_fN_ compared to tools with continuous cutting edges—by an average of up to 57%. However, at higher radial infeed *a_e_*, tools with modified edge geometries exhibited a notable increase in signal variability—up to 300%—suggesting the excitation of resonance phenomena within the MHTW system.
- Serrated and wavy tools generated higher maximum and average values of the *F_a_* and *F_f_* force components at low *f_z_* (up to 101% increase for *F_f_* at *f_z_* = 0.05 mm/tooth), while at higher *f_z_*, the continuous-edge cutter produced greater force values.
- Increasing radial depth of cut *a_e_* resulted in a monotonic growth of all cutting-force components for all tool geometries. However, the dispersion of the *F_fN_* component at *a_e_* = 10 mm was markedly higher for serrated (by ~300%) and wavy tools (by ~160%) compared to the continuous-edge tool.
- The axial force component (*Fa*) demonstrated the highest effectiveness in distinguishing tools with continuous cutting edges from those with wavy and serrated profiles, as confirmed by Linear Discriminant Analysis.
- Recurrence Quantification Analysis (RQA) proved to be a valuable tool for quantitatively assessing the dynamic complexity and stability of the milling process. Indicators such as DET, L, LAM, and TT exhibited strong sensitivity to changes in tool edge geometry and machining parameters.
- Continuous-edge tools consistently displayed the highest DET, LAM and L values across all tested cutting speeds, indicating a more ordered, predictable, and stable cutting process. Serrated and wavy tools exhibited approximately 30% lower values of the DET and LAM indicators compared to the continuous-edge tool, indicating a degradation of regularity and the emergence of chaotic behavior in the cutting-force signal.
- The continuous-edge tool exhibited the highest values of DET, LAM, L, and TT across the analyzed *fz* range, indicating superior dynamic stability and order, especially at *fz* = 0.1 mm/tooth. Serrated and wavy tools showed approximately 30–40% lower values of these indicators, though their stability improved slightly with increasing *fz*.
- The continuous-edge tool exhibited the highest and most stable values of DET (>0.9), LAM (>0.9), L (~5), TT (>4.5), and Vmax (~11–13) across the full range of radial infeed *ae*, confirming its superior ability to maintain regular and stable dynamic behavior under increasing cutting loads. In contrast, serrated and especially wavy geometries showed significant decreases in these indicators with increasing *ae*—for example, wavy-edge tools showed a drop in DET from ~0.65 to ~0.5, LAM from ~0.65 to ~0.15, and Vmax from ~17 to ~4—indicating rising instability and irregularity in the process.

## Figures and Tables

**Table 1:** Two factor experiment plan: technological parameter versus type of the cutting edge.

Stage of Research	Constant Parameters	Cutting Speed *v_c_* m/min	Shape of the Cutting Edge
Stage I	Feed per tooth *f_z_*, 0.075 mm/z Axial infeed *a_p_*, 10 mm Radial infeed *a_e_*, 3 mm	200	Continues
		400	Serrated
		600	Wavy
		**Feed per tooth *f_z_*** **mm/z**	
Stage II	Cutting speed *v_c_*, 500 m/min Radial infeed *a_e_*, 3 mm Axial infeed *a_p_*, 10 mm	0.05	Continues
		0.1	Serrated
		0.15	Wavy
		**Radial infeed *a_e_*** **mm**	
Stage III	Cutting speed *v_c_*, 500 m/min Feed per tooth *f_z_*, 0.075 mm/z Axial infeed *a_p_*, 10 mm	4	Continues
		7	Serrated
		10	Wavy

**Table 2:** Endmill cutters specification.

Endmill Cutter	Photo	Shape of the Cutting Edge	Helix Angle λ °	Total Length L mm	Flute Length L_f_ mm
3ALE 120 350 080	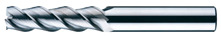				
		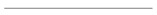	45	80	25
			
3ARO 120 240 S12	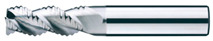		45		
		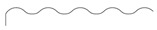		80	24
		
3ARE 120 300 S12	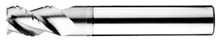		45		
		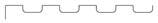		80	20
		

**Table 3:** RQA indicators for different cutting speed *v_c_* values and cutting-edge types for all cutting-force components.

*v_c_*	Tool	DET	Lmax	L	DIV	ENTR	LAM	TT	Vmax	RPDE	TRANS	CC	Cutting Force Component
m/min	Type	-	-	-	-	-	-	-	-	-	-	-	-
200	C	0.966	446	6.301	2.24 × 10^−3^	3.153	0.984	7.095	20	0.864	0.716	0.732	F_a_
200	C	0.969	488	7.434	2.05 × 10^−3^	3.502	0.984	4.653	17	0.869	0.634	0.657	F_f_
200	C	0.982	470	7.177	2.13 × 10^−3^	3.256	0.993	7.050	25	0.851	0.660	0.694	F_fN_
400	C	0.940	466	5.143	2.15 × 10^−3^	2.500	0.970	4.455	12	0.839	0.627	0.668	F_a_
400	C	0.991	470	9.571	2.13 × 10^−3^	4.076	0.885	2.249	4	0.882	0.720	0.720	F_f_
400	C	0.983	492	9.785	2.03 × 10^−3^	3.836	0.980	3.481	8	0.852	0.669	0.694	F_fN_
600	C	0.978	458	8.593	2.18 × 10^−3^	3.777	0.962	2.874	7	0.856	0.725	0.723	F_a_
600	C	0.988	494	15.671	2.02 × 10^−3^	4.107	0.976	2.613	5	0.900	0.744	0.738	F_f_
600	C	0.987	486	16.783	2.06 × 10^−3^	3.634	0.991	2.537	4	0.875	0.756	0.749	F_fN_
200	S	0.904	473	4.720	2.11 × 10^−3^	2.485	0.942	3.293	11	0.848	0.592	0.627	F_a_
200	S	0.942	482	5.111	2.07 × 10^−3^	2.730	0.969	4.556	17	0.895	0.550	0.608	F_f_
200	S	0.938	404	4.346	2.48 × 10^−3^	2.485	0.972	3.933	13	0.866	0.561	0.636	F_fN_
400	S	0.569	488	3.116	2.05 × 10^−3^	1.429	0.632	2.348	9	0.905	0.600	0.648	F_a_
400	S	0.899	488	3.907	2.05 × 10^−3^	1.860	0.927	2.900	7	0.865	0.681	0.694	F_f_
400	S	0.839	486	4.087	2.06 × 10^−3^	1.727	0.918	3.053	9	0.849	0.647	0.674	F_fN_
600	S	0.500	491	5.739	2.04 × 10^−3^	2.054	0.649	2.547	7	0.871	0.593	0.676	F_a_
600	S	0.948	494	10.010	2.02 × 10^−3^	3.634	0.975	2.939	6	0.879	0.730	0.722	F_f_
600	S	0.941	488	5.358	2.05 × 10^−3^	2.653	0.953	2.818	5	0.861	0.745	0.737	F_fN_
200	W	0.808	470	3.765	2.13 × 10^−3^	2.215	0.919	3.584	13	0.867	0.625	0.670	F_a_
200	W	0.921	425	4.486	2.35 × 10^−3^	2.495	0.969	4.505	17	0.872	0.601	0.662	F_f_
200	W	0.924	470	5.763	2.13 × 10^−3^	2.804	0.966	3.771	15	0.845	0.604	0.642	F_fN_
400	W	0.556	492	3.398	2.03 × 10^−3^	1.578	0.712	2.661	12	0.892	0.616	0.657	F_a_
400	W	0.915	477	4.808	2.10 × 10^−3^	2.874	0.896	2.572	6	0.883	0.704	0.715	F_f_
400	W	0.928	434	6.048	2.30 × 10^−3^	3.102	0.939	3.869	10	0.828	0.707	0.719	F_fN_
600	W	0.617	496	5.628	2.02 × 10^−3^	3.018	0.707	2.547	7	0.853	0.646	0.683	F_a_
600	W	0.944	468	7.008	2.14 × 10^−3^	3.025	0.957	3.629	9	0.866	0.672	0.686	F_f_
600	W	0.973	490	8.583	2.04 × 10^−3^	3.276	0.963	2.680	5	0.878	0.728	0.729	F_fN_

**Table 4:** RQA indicators for different feed per tooth *f_z_* values and cutting-edge types for all cutting-force components.

*f_z_*	Tool	DET	Lmax	L	DIV	ENTR	LAM	TT	Vmax	RPDE	TRANS	CC	Cutting-Force Component
mm/z	Type	-	-	-	-	-	-	-	-	-	-	-	-
0.05	C	0.818	486	4.393	2.06 × 10^−3^	1.820	0.939	3.959	13	0.848	0.673	0.736	F_a_
0.05	C	0.930	488	5.276	2.05 × 10^−3^	2.397	0.960	3.499	9	0.868	0.677	0.702	F_f_
0.05	C	0.988	492	9.676	2.03 × 10^−3^	3.738	0.988	4.234	12	0.877	0.676	0.693	F_fN_
0.1	C	0.916	494	5.687	2.02 × 10^−3^	2.788	0.937	2.998	12	0.854	0.641	0.675	F_a_
0.1	C	0.908	492	5.252	2.03 × 10^−3^	2.513	0.953	3.192	12	0.870	0.608	0.645	F_f_
0.1	C	0.972	490	10.579	2.04 × 10^−3^	3.672	0.987	3.681	9	0.876	0.720	0.722	F_fN_
0.15	C	0.843	474	4.813	2.11 × 10^−3^	2.461	0.848	2.467	7	0.829	0.622	0.669	F_a_
0.15	C	0.845	476	3.917	2.10 × 10^−3^	2.116	0.804	2.316	6	0.871	0.612	0.639	F_f_
0.15	C	0.957	490	10.122	2.04 × 10^−3^	3.331	0.968	3.303	8	0.862	0.732	0.720	F_fN_
0.05	S	0.622	480	3.828	2.08 × 10^−3^	1.917	0.695	2.468	9	0.876	0.640	0.669	F_a_
0.05	S	0.677	492	4.111	2.03 × 10^−3^	1.722	0.861	2.888	12	0.882	0.625	0.670	F_f_
0.05	S	0.756	490	3.819	2.04 × 10^−3^	1.648	0.899	3.152	11	0.863	0.658	0.695	F_fN_
0.1	S	0.627	480	3.851	2.08 × 10^−3^	1.970	0.748	2.606	9	0.873	0.668	0.703	F_a_
0.1	S	0.769	490	4.154	2.04 × 10^−3^	1.791	0.897	3.136	9	0.862	0.663	0.692	F_f_
0.1	S	0.855	484	4.048	2.07 × 10^−3^	2.024	0.926	3.418	9	0.863	0.691	0.703	F_fN_
0.15	S	0.656	490	3.721	2.04 × 10^−3^	1.518	0.851	2.767	6	0.866	0.671	0.703	F_a_
0.15	S	0.865	494	4.789	2.02 × 10^−3^	2.449	0.900	3.157	10	0.871	0.627	0.664	F_f_
0.15	S	0.846	494	5.195	2.02 × 10^−3^	2.550	0.926	3.391	10	0.873	0.667	0.693	F_fN_
0.05	W	0.552	480	3.983	2.08 × 10^−3^	2.122	0.622	2.294	5	0.876	0.598	0.649	F_a_
0.05	W	0.744	467	3.874	2.14 × 10^−3^	1.630	0.887	3.086	7	0.865	0.603	0.678	F_f_
0.05	W	0.624	480	3.973	2.08 × 10^−3^	1.819	0.824	2.657	7	0.876	0.637	0.691	F_fN_
0.1	W	0.643	454	3.479	2.20 × 10^−3^	1.752	0.774	2.643	7	0.856	0.667	0.691	F_a_
0.1	W	0.848	488	4.767	2.05 × 10^−3^	2.293	0.913	3.196	9	0.886	0.635	0.668	F_f_
0.1	W	0.854	488	4.735	2.05 × 10^−3^	2.332	0.934	3.189	8	0.836	0.683	0.700	F_fN_
0.15	W	0.638	479	3.809	2.09 × 10^−3^	1.876	0.723	2.436	7	0.861	0.588	0.634	F_a_
0.15	W	0.858	494	5.184	2.02 × 10^−3^	2.678	0.911	3.113	9	0.876	0.674	0.690	F_f_
0.15	W	0.667	484	3.822	2.07 × 10^−3^	1.735	0.824	2.932	8	0.849	0.672	0.724	F_fN_

**Table 5:** RQA indicators for different radial infeed *a_e_* values and cutting-edge types for all cutting-force components.

*a_e_*	Tool	DET	Lmax	L	DIV	ENTR	LAM	TT	Vmax	RPDE	TRANS	CC	Cutting Force Component
mm	Type	-	-	-	-	-	-	-	-	-	-	-	-
4	C	0.929	478	5.168	2.09 × 10^−3^	2.428	0.980	4.397	10	0.844	0.704	0.731	F_a_
4	C	0.912	440	4.738	2.27 × 10^−3^	2.401	0.966	3.868	9	0.847	0.690	0.722	F_f_
4	C	0.980	432	7.704	2.31 × 10^−3^	3.294	0.981	3.550	8	0.847	0.705	0.709	F_fN_
7	C	0.932	478	5.076	2.09 × 10^−3^	2.480	0.969	4.762	12	0.839	0.716	0.732	F_a_
7	C	0.960	456	5.667	2.19 × 10^−3^	2.787	0.976	3.847	10	0.856	0.725	0.714	F_f_
7	C	0.986	484	10.586	2.07 × 10^−3^	3.836	0.986	3.749	8	0.872	0.711	0.707	F_fN_
10	C	0.801	468	4.155	2.14 × 10^−3^	2.047	0.928	3.568	10	0.856	0.627	0.675	F_a_
10	C	0.883	476	5.184	2.10 × 10^−3^	2.379	0.968	3.788	10	0.832	0.683	0.713	F_f_
10	C	0.952	488	5.956	2.05 × 10^−3^	2.607	0.978	4.224	9	0.862	0.687	0.688	F_fN_
4	S	0.672	492	4.512	2.03 × 10^−3^	1.902	0.798	2.671	8	0.848	0.671	0.706	F_a_
4	S	0.725	494	4.810	2.02 × 10^−3^	2.322	0.860	2.846	13	0.892	0.604	0.661	F_f_
4	S	0.825	468	4.029	2.14 × 10^−3^	2.139	0.921	3.492	8	0.856	0.695	0.708	F_fN_
7	S	0.736	488	4.644	2.05 × 10^−3^	1.795	0.864	2.979	10	0.847	0.615	0.654	F_a_
7	S	0.862	488	4.329	2.05 × 10^−3^	2.141	0.939	3.655	11	0.869	0.660	0.691	F_f_
7	S	0.935	496	7.514	2.02 × 10^−3^	3.374	0.963	3.239	9	0.820	0.712	0.716	F_fN_
10	S	0.697	492	3.736	2.03 × 10^−3^	2.088	0.021	2.000	2	0.860	0.680	0.695	F_a_
10	S	0.657	488	3.482	2.05 × 10^−3^	1.872	0.661	2.955	13	0.874	0.580	0.622	F_f_
10	S	0.597	492	3.545	2.03 × 10^−3^	1.772	0.111	2.010	3	0.871	0.607	0.632	F_fN_
4	W	0.646	491	4.498	2.04 × 10^−3^	2.354	0.668	2.849	17	0.874	0.558	0.603	F_a_
4	W	0.737	449	3.655	2.23 × 10^−3^	1.845	0.852	2.891	8	0.868	0.569	0.629	F_f_
4	W	0.694	486	3.580	2.06 × 10^−3^	2.032	0.818	2.880	9	0.866	0.630	0.660	F_fN_
7	W	0.595	482	3.287	2.07 × 10^−3^	1.922	0.137	2.007	3	0.862	0.583	0.624	F_a_
7	W	0.647	479	3.029	2.09 × 10^−3^	1.779	0.773	3.075	10	0.871	0.609	0.646	F_f_
7	W	0.579	484	3.812	2.07 × 10^−3^	2.245	0.708	2.527	8	0.859	0.615	0.647	F_fN_
10	W	0.518	492	2.977	2.03 × 10^−3^	1.486	0.167	2.053	4	0.869	0.598	0.637	F_a_
10	W	0.622	460	2.872	2.17 × 10^−3^	1.700	0.745	2.938	10	0.887	0.646	0.664	F_f_
10	W	0.509	488	3.213	2.05 × 10^−3^	1.813	0.404	2.103	6	0.904	0.596	0.630	F_fN_

**Table 6:** RP plots as a function of cutting speed and cutting-edge type for the axial component of the cutting force F_a_.

*v_c_* m/min	Continuos	Serrated	Wavy
200	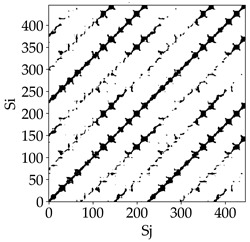	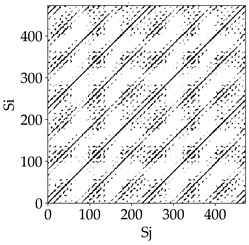	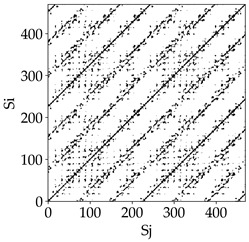
400	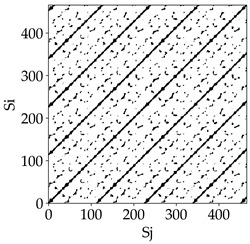	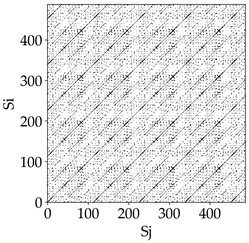	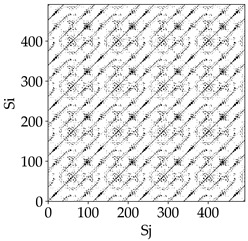
600	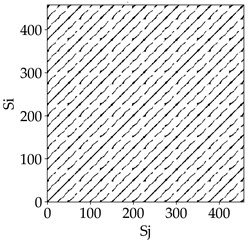	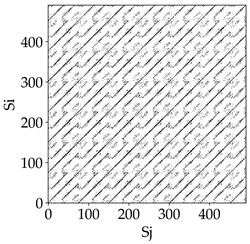	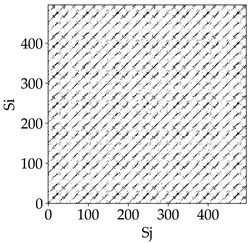

**Table 7:** RP plots as a function of feed per tooth and cutting-edge type for the axial component of the cutting force F_a_.

*f_z_* mm/z	Continuos	Serrated	Wavy
0.05	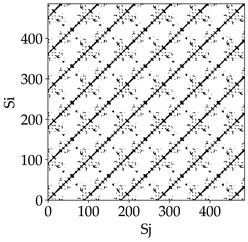	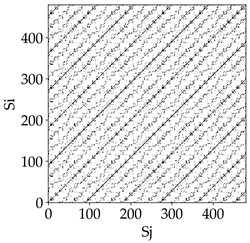	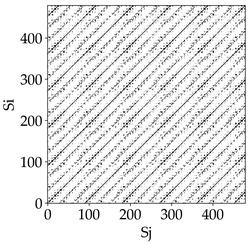
0.1	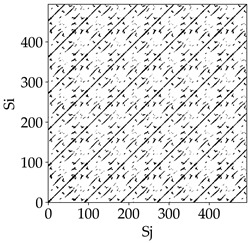	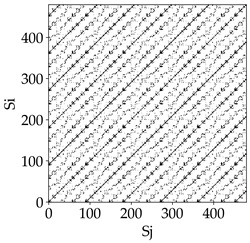	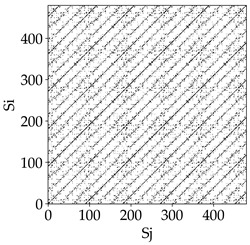
0.15	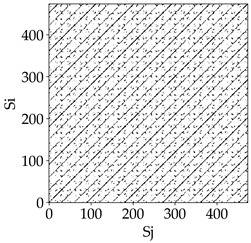	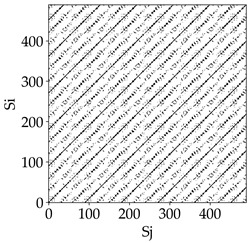	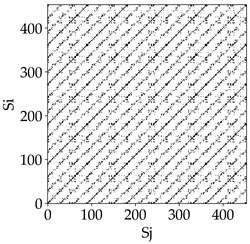

**Table 8:** RP plots as a function of radial infeed and cutting-edge type for the axial component of the cutting-force F_a_.

*a_e_* mm	Continuos	Serrated	Wavy
4	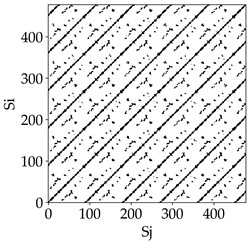	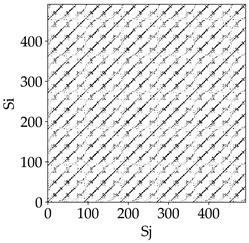	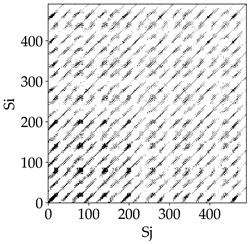
7	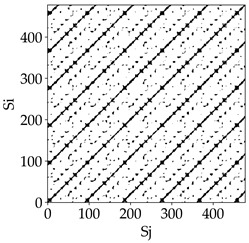	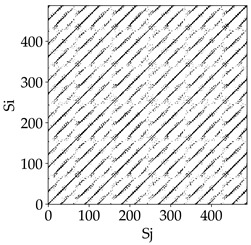	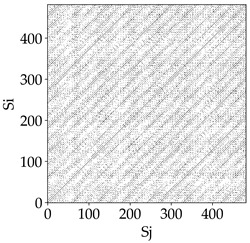
10	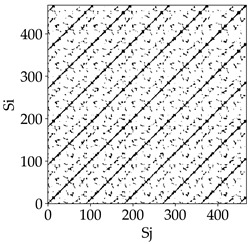	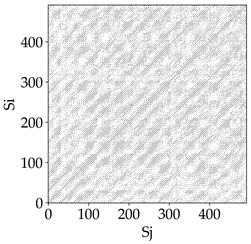	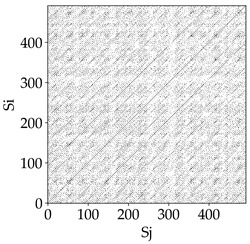

## Data Availability

The original contributions presented in this study are included in the article. Further inquiries can be directed at the corresponding author.

## Abbreviations

The following abbreviations are used in this manuscript:

CAD	Computer Aided Drawing
CAM	Computer Aided Manufacturing
MQL	Minimum Quantity Lubrication
HPC	High-Performance Cutting
CRQA	Cross Recurrence Quantification Analysis
LDA	Linear Discriminant Analysis
MHTW	Machine–Holder–Tool–Workpiece system
RAQ	Recurrence Quantification Analysis
RP	Recurrence plot
DET	Determinism
L	Average Diagonal Line Length
Lmax	Longest Diagonal Line
DIV	Divergence
ENTR	Entropy
TT	Trapping Time
Vmax	Max Vertical Line
RPDE	Recurrence Period Density Entropy
TRANS	Transition Rate
CC	Clustering Coefficient
